# Supplementary material for: Chromothripsis during telomere crisis is independent of NHEJ, and consistent with a replicative origin
Source: Genome Res. 2019 May;29(5):737–49. doi: 10.1101/gr.240705.118 (PMC6499312; doi:10.1101/gr.240705.118)
Supplement: Supplemental Material [file supp_gr.240705.118_Supplemental_file_1.zip › contigs/annotated_contigs/DB93/contig.2.DB93_length_427_mean_cov_3.86416861827.docx]

**DB93_length_427_mean_cov_3.86416861827**

```
AGTAGAAAGTTTTGCTTTACTAACACCTGAAGAATGATACAATCACAGGCAATCTGGTTTTTATATGTTGTATTACATTAAAGTAAAAG
 >chr17:57808039-57808200 + E=2e-82
CACTGATACCTGTAGTATGAAATGAAGCATGTTATAAATAAAGTACATTTAACTATCTTAAACAT|CTCAAGT|GATCCACGCACCTCC
                                                                  >chr17:57810572-57810
GCCTCCCAAAGTGCTGGGATTACAGATGTGAGCCACCACGCCCAGCACAAAATGCATATACACTGTAAACCGGTAATTCCATTTCTAGG
845 - E=6e-153
AATGATTAGGTATAAGGTGAAATGACTTGCACAACTTTATTGACCACAGCATTATTTGTACTAGCAAAAAATAATCAATAACTTATATA

TTACCAATAGAGGACAGAAGAGTGATTAAAGTACAGCACACTCGTACAATGGAAAGAAAAACTAAGCAGCCAT
```
